# Maternal media exposure and child anthropometric failures across 40 low- and middle-income countries

**DOI:** 10.1016/j.ssmph.2024.101746

**Published:** 2024-12-31

**Authors:** Sohee Jung, Hwa-Young Lee, Seung-ah Choe, Hannah Oh, S.V. Subramanian, Rockli Kim

**Affiliations:** a Interdisciplinary Program in Precision Public Health, Department of Public Health Sciences, Graduate School of Korea University, 145 Anam-ro, Seongbuk-gu, Seoul, 02841, Republic of Korea; b Department of Global Health, Graduate School of Public Health and Healthcare Management, The Catholic University of Korea, 222 Banpo-daero, Seocho-gu, Seoul, 06591, Republic of Korea; c Department of Preventive Medicine, Korea University College of Medicine, 73 Anam-ro, Seongbuk-gu, Seoul, 02841, Republic of Korea; d Division of Health Policy and Management, College of Health Sciences, Korea University, 145 Anam-ro, Seongbuk-gu, Seoul, 02841, Republic of Korea; e Harvard Center for Population and Development Studies, 9 Bow Street, Cambridge, MA, 02138, USA; f Department of Social and Behavioral Sciences, Harvard T.H. Chan School of Public Health, 677 Huntington Avenue, Boston, MA, 02115, USA

**Keywords:** Media exposure, Newspaper/magazines, Television, Radio, Mobile phone, Internet, Child anthropometric failures, Demographic and health survey, Low- and middle-income countries

## Abstract

**Objective:**

To examine the association between maternal exposure to various types of media and child anthropometric failures, and whether this association varies by mothers’ socioeconomic status (SES) in low- and middle-income countries (LMICs).

**Methods:**

This study analyzed Demographic and Health Survey data from 40 LMICs (2010–2024). The independent variable was defined as exposure to any mass media (i.e., newspapers/magazines, radio, television) at least once a week for the primary analysis, and mobile phone ownership and internet access were considered for secondary analyses. Three types of child anthropometric failures (i.e., stunting, underweight, wasting) were constructed based on the WHO child growth standards. Multivariable logistic regression models were conducted to examine the association between maternal media exposure and child anthropometric failures, as well as the moderating effects of mothers’ socioeconomic status.

**Results:**

Among 439,639 children aged under five, 13% were stunted, 23% were underweight, and 13% had wasting. Overall, 53% of mothers were exposed to any mass media, which was significantly associated with reduced odds of child stunting (OR = 0.90, 95% CI = 0.88–0.92), underweight (OR = 0.89, 95% CI = 0.87–0.91), and wasting (OR = 0.93, 95% CI = 0.90–0.96). The same was true for maternal mobile phone ownership and internet access. For specific types of media, the association was more pronounced among uneducated women and wealthier households.

**Conclusions:**

This study underscores the potential importance of media-based interventions in improving child undernutrition in LMICs. Media exposure is an important channel for health information dissemination and empowerment especially for women with no education. At the same time, improvements in the overall living standards are necessary to induce and sustain healthy behaviors to ensure optimal growth among children.

## Highlights

• Maternal mass media exposure, mobile phone ownership, and internet access were significantly associated with lower odds of child anthropometric failures.
• Media exposure significantly reduced the risk of child anthropometric failures among uneducated women but not necessarily for higher educated groups.
• Exposure to radio and mobile phone ownership was more strongly associated with reduction in child anthropometric failures among wealthier households.
• This study underscores the potential importance of media-based interventions in improving child undernutrition in LMICs.

## 1 Introduction

Child undernutrition – defined as a pathological state resulting from inadequate intake of energy and other nutrients – contributes to half of all under-five mortalities worldwide (United Nations Children's Fund, 2023). As per anthropometric failure indicators, 149 million children worldwide suffered from stunting (i.e., low height-for-age) and 45 million from wasting (i.e., low weight-for-height) in 2022 (United Nations Children's Fund, 2023), of which about 68% occurred in low- and middle-income countries (LMICs) (United Nations Children’s Fund UNICEF & World Health Organization WHO, 2023). Inadequate nutrition and growth in early childhood are associated with poor cognitive development, low educational performance, and low economic productivity in adulthood (Horton & Steckel, 2013; Perkins et al., 2017). Therefore, evidence-based, effective interventions are needed to prevent child undernutrition.

The United Nations International Children's Emergency Fund (UNICEF) framework on determinants of maternal and child nutrition outlines adequate food and dietary practices and good care as two immediate determinants of child undernutrition (United Nations Children’s Fund, 2020). For example, appropriate feeding practices can directly prevent and treat undernutrition by meeting the nutritional requirements of children in their developmental period (World Health Organizaion, 2023). However, children’s adherence to appropriate feeding practices remains far inadequate. According to a study based on 92 LMICs, 10% of children aged 6–23 months were found not to have been fed of any animal milk, formula, or solid or semisolid food within 24 h (Karlsson et al., 2024). Importantly, these health behaviors related to child nutrition are usually performed by caregivers, especially mothers (Caruso et al., 2010; Ekbrand & Halleröd, 2018; Kraamwinkel et al., 2019). For this reason, maternal characteristics related to their abilities to utilize, direct, and control resources can significantly impact their children’s health, safety, and nutrition (Kabir, 2022). Thus, fostering mothers’ awareness of nutrition-related information and proper healthcare utilization can enhance their engagement in healthy practices and ultimately improve growth and development among children in LMICs.

In this regard, various forms of media can be effective channels to disseminate health-related information (Dutta-Bergman, 2004), but their distinct characteristics can influence individual’s health behaviors and outcomes differently (Viswanath et al., 2015, pp. 327–348). Mass media generates and disseminates information to vast, undifferentiated audiences, and they can play important roles in defining health issues and disseminating the latest health information to sustain attention and lead audiences to perceive those issues as necessary (Viswanath et al., 2007). Therefore, the widespread reach of mass media, including newspapers, radio, and television, can effectively distribute health-related knowledge and information to diverse audiences and help them make appropriate decisions about their health (Fatema & Lariscy, 2020; Hong & Kim, 2020; Kabir, 2022). In particular, newspapers provide health-related information through textual data, which is especially effective for those who can read and process information in written form (Fritz, 2001), while television utilizes imagery and audiovisual effects to convey health messages (Robinson et al., 2014). In some child health improvement programs, radio dramas have been used to provide health-related information through audio (Saaka et al., 2021).

At the same time, the rapid growth in other types of media (e.g., mobile phones and internet connectivity) enable direct and continuous communication (World Bank, 2012), which allow women to be empowered and connected to information and health services in LMICs (Women Connected, 2019; Yepes et al., 2016). For example, mobile messages by health personnel were found to be effective in improving mothers’ child-feeding practices in LMICs (Jerin et al., 2020).

To date, studies assessing the role of maternal media exposure have largely focused on behavioral change outcomes related to child undernutrition (Alexander et al., 2019; Beckstead et al., 2022; Hanson et al., 2020; Jerin et al., 2020; Kim et al., 2019; Menon et al., 2016; Rawat et al., 2017; Saaka et al., 2021; Suresh et al., 2019). For instance, in three experimental studies, mother’s exposure to media was found to have a consistently significant effect on improving complementary feeding, minimum dietary diversity, and minimum acceptable diet among infants and young children (Kim et al., 2019; Menon et al., 2016; Saaka et al., 2021).

At the same time, evidence on the association between maternal media exposure and child anthropometric failure in LMICs remain mixed. Two previous studies found that delivering specific messages to encourage appropriate feeding practices via mobile phones can significantly lower the risk of child anthropometric failures in Bangladesh and Cambodia (George et al., 2021; Young et al., 2021). However, a study conducted in Ghana revealed that after 12 months of nutrition-related messages through radio intervention, no significant difference was found in child anthropometric status in the intervention group compared with the control group (Saaka et al., 2021). Hence, there is a need for a more comprehensive assessment of the relationship between maternal media exposure and child anthropometric failure outcomes.

Furthermore, only a few studies have considered potential heterogeneities in this association by media types or by mothers’ socioeconomic status (SES) (Dhawan et al., 2023; Kabir, 2022). Individuals with higher education are more likely to have better health literacy (Paasche-Orlow et al., 2005) to convert health-related information into health behaviors and to effectively distribute resources to obtain better healthcare than less educated people (Cowell, 2006). Text-based media, in particular, tends to be more effective for educated people who are literate and familiar with complex vocabulary and excel in interpreting textual information (Leroy et al., 2006). Therefore, different characteristics of media types should be considered when evaluating their health impacts, especially across different SES groups.

Consequently, this study sought to examine the association between maternal exposure to various types of media and child anthropometric failure outcomes in LMICs, and whether there are heterogeneities in theses associations by SES. Based on theoretical background and previous evidence, we expected mother’s exposure to mass media (i.e., newspapers or magazines, television, and radio), mobile phone ownership, and internet access to be associated with a lower likelihood of child anthropometric failure. Secondly, we hypothesized that maternal education, household wealth, and place of residence would significantly moderate this association.

## 2 Methods

### 2.1 Data source

Demographic and Health Survey (DHS) provides nationally representative health information, including nutrition and disease indicators, from over 90 LMICs every five years (International Institute for Population Sciences, 2022). A typical survey design in DHS follows a two-stage cluster sampling frame, such that primary sampling units (PSUs) from rural areas and census enumeration blocks in urban areas are selected in the first stage. The second stage involves selection of households from each PSU with an equal probability systematic sampling. More detailed descriptions of the survey design can be found elsewhere (International Institute for Population Sciences, 2022).

### 2.2 Study population

Based on the World Bank Country Classification in 2023, we identified 49 low- and lower-middle-income countries with the most recent DHS collected after 2010 to ensure comparability and consistency in measurements. Among 49 countries, we excluded 9 countries with absent data on child anthropometric failures and/or maternal mass media exposure: Afghanistan, Congo, Ethiopia, Haiti, Honduras, Madagascar, Mauritania, Papua New Guinea, and the Philippines. Thereby, this study included 40 countries in the main analysis. Data collection overlapped with the COVID-19 pandemic (2020–2022) in nine countries, including Burkina Faso, Cambodia, Cote d’Ivoire, Gambia, Ghana, India, Liberia, Nepal, and Rwanda.

**Fig. 1:**
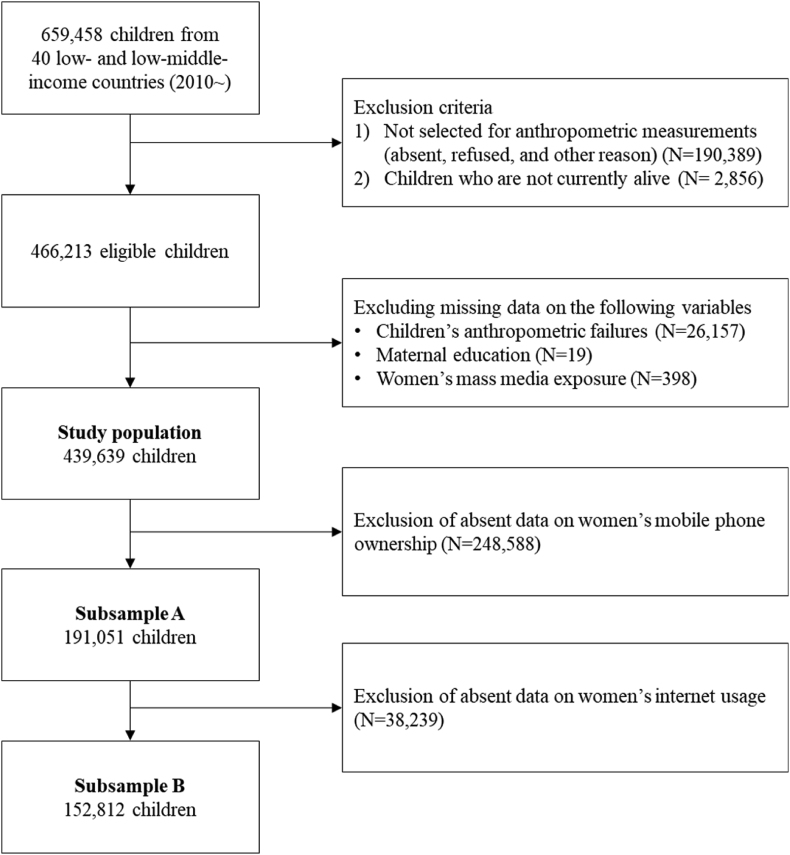
Fow diagram for study population.

Across these selected countries, 659,458 children under five were surveyed, among which 466,213 children were selected for random anthropometric measurements and were alive at the time of the survey (Fig. 1). Among 466,213 eligible children, those with missing or implausible anthropometric measures, defined as height-for-age z-score (HAZ) below or above 6 standard deviations (SD), weight-for-age z-score (WAZ) below 6 SD or above 5 SD, and weight-for-height z-score (WHZ) below or above 5 SD of the median of the WHO Child Growth Standards (World Health Organization, 2006), were excluded (5.61%). Further excluding observations with missing data on maternal education level (0.004%) and maternal mass media exposure (0.09%), the final analytic sample consisted of 439,639 children. Of note, when the original sample (N = 659,458) and the final analytic sample were compared on basic demographic and socioeconomic characteristics, minimum differences were found (Supplementary Table 1).

For the secondary analysis on mobile phone ownership and internet access, subsamples were used due to absence of data on mothers’ mobile phone ownership and internet access in some countries: 191,051 children in 28 countries (subsample A) and 152,812 children in 26 countries (subsample B).

### 2.3 Dependent variable

Three anthropometric failure outcomes were used to assess child undernutrition: stunting, underweight, and wasting. Child stunting refers to retarded skeletal growth resulting from chronic or recurrent nutrition deprivation, while wasting indicates an acute form of undernutrition. Underweight is regarded as a composite measure for both acute and chronic undernutrition (Caulfield et al., 2006). Trained field staffs in DHS utilized Shorr Boards to measure the length of children under 2 years lying down and the height of children over 2 years standing up, with weight being measured using a SECA digital scale (Assaf et al., 2015). Based on the WHO Child Growth Standards, children were defined as stunting, underweight, and wasting if their HAZ, WAZ, and WHZ were −2 SD below the median values, respectively (World Health Organization, 2006).

### 2.4 Independent variables

For the primary analysis, maternal mass media exposure was defined as mothers’ exposure to one of the following at least once a week or daily: newspapers/magazines, radio, and television. Frequency of exposure to newspapers/magazines, radio, and television within the week were asked, respectively, using three questions: “Do you read a newspaper or magazine at least once a week, less than once a week, or not at all?”, “Do you listen to the radio at least once a week, less than once a week, or not at all?” and “Do you watch television at least once a week, less than once a week, or not at all?”. Based on the responses, a binary indicator was constructed following the DHS guideline with ‘1’ coded for mothers if they read newspapers/magazines, listened to radio, or watched television at least once a week and ‘0’ otherwise.

Two additional variables were considered for secondary analysis. For mobile phone ownership, mothers were asked: “Do you own a mobile telephone?”. A binary indicator was constructed with ‘1’ indicating mothers who owned a mobile phone and ‘0’ otherwise. Internet access was defined as using the internet at least once within the last month. Mothers were asked, “How often did you use the internet?”. Based on their responses, a binary indicator was constructed, with ‘1’ coded for mothers who utilized the internet at least once a week or almost every day and ‘0’ otherwise.

### 2.5 Control variables

Based on the review of existing literature, this study included the following demographic and socioeconomic characteristics as control variables: maternal age (15–19 years; 20–34 years; 35–49 years), marital status (not married; married), age of child (0–24 months; 25–60 months), sex of child (male; female), maternal education level (no education; primary; secondary; higher), household wealth quintiles (poorest; poorer; middle; richer; richest), and type of residence (rural; urban). We constructed a binary variable for child’s age to reflect the higher risk of undernutrition and infection in the first two years of life (Karlsson et al., 2022). Four categories of maternal educational level were constructed following the DHS standardized education variables to ensure comparability across countries. The DHS wealth variable categorizes households into quintiles based on a composite measure of households’ cumulative living standards constructed from household’s ownership of consumer items (e.g., television and car) as well as dwelling characteristics (e.g., flooring materials) (Rutstein & Johnson, 2004).

### 2.6 Statistical analysis

Separate analyses were conducted for mothers’ exposure to mass media (main study sample; N = 439,639), mobile phone ownership (subsample A; N = 191,051), and internet access (subsample B; N = 152,812). This study first examined the overall and country-specific prevalence of three anthropometric failures and maternal media exposure. Additionally, the distribution of the main study sample and the outcomes were explored by each of the predictors. Sampling weights were included in all descriptive analyses to account for the differential probabilities of participation and selection.

Then, we estimated a series of multivariable logistic regression models to evaluate the association between media exposure and three child anthropometric failures. Specifically, the probability of child anthropometric failure $\left(Y_{i}=1\right)$ was modelled as:

(1) $$\text{Logit}\;\left(\mathrm{Pr}\left(Y_{i}=1\;|X\right)\right)={\;\beta }_{0}+{\;\beta }_{1}\text{Media}\;\text{exposure}+{\;\beta }_{2}X_{i}^{\prime }+{\;\beta }_{3}C+\varepsilon_{0i}$$

Model 1 adjusted for all demographic and socioeconomic covariates $\left(X_{i}^{\prime }\right)$ and controlled for country fixed effects (C) (Eq. (1)).

To assess heterogeneity by maternal SES (i.e., maternal education level, household wealth, and type of residence), stratified analyses were performed. In addition, we added an interaction term between maternal media exposure and maternal SES in Eq. (1) to test for significant heterogeneity in their relationship with child anthropometric failures. We conducted likelihood ratio (LR) tests to obtain the statistical significance of the interaction terms.

We also conducted sensitivity analyses to enhance the robustness of our findings. First, sensitivity analyses were performed with different classifications of mass media exposure (categorical: not at all, less than once a week, at least once a week, daily) to explore potential dose-response relationships. Second, country-specific analyses were performed to assess heterogeneity in the relationship between maternal mass media exposure and child anthropometric failures across countries. Lastly, to remove potential COVID-19 effect, we re-ran the analyses after excluding nine countries where data collection overlapped with the COVID-19 pandemic (i.e., Burkina Faso, Cambodia, Cote d’Ivoire, Gambia, Ghana, India, Liberia, Nepal, and Rwanda).

All analyses were performed using STATA version 16 (StataCorp, 2019). The significance level for all statistical analyses was established at P < 0.05, using two-tailed tests. The multivariable analysis results were reported with odds ratios (ORs) and 95% confidence intervals (CIs).

## 3 Results

Of the primary analytic sample of 439,639 children, 33.05% were stunted, 23.21% were underweight, and 12.85% had wasting (Table 1). Regarding mass media exposure, 52.95% of mothers were identified as having any mass media exposure: reading newspapers/magazines, listening to the radio, or watching a television. By different types of mass media, television watching (43.23%) was most prevalent, followed by listening to radio (18.12%) and reading newspapers or magazines (8.44%). Based on subsample A and subsample B, the overall proportion of mothers who had mobile phones and internet access was 57.59% and 14.87%, respectively. Mothers with stunted children were less likely to be exposed to any mass media (57.35% vs. 44.05%) (Supplementary Table 2). Likewise, mothers with underweight children had less access to mass media than mothers whose children were not underweight (56.12% vs. 42.48%), which was also true for mothers with children having wasting (53.79% vs. 47.27%).

**Table 1:** Descriptive summary of child anthropometric failures, maternal mass media exposure, mobile phone ownership, and internet access across low- and middle-income countries (%).

			Child anthropometric failures			Mass media exposure				Mobile phone ownership	Internet access
Survey year		N	Stunting	Underweight	Wasting	Any media	Newspaper/Magazine	Radio	Television		
Pooled		439,639	33.05	23.21	12.85	52.95	8.44	18.12	43.24	57.59	14.87
Angola	2015–16	6268	37.30	18.55	5.03	66.28	15.71	49.65	55.71	40.17	5.87
Bangladesh	2017–18	7806	31.32	22.14	8.46	54.31	2.72	2.06	53.46	62.67	NA
Benin	2017–18	11,631	31.73	16.51	5.18	41.23	2.25	35.12	18.04	50.46	3.17
Burkina Faso	2021	5712	21.20	16.18	9.91	50.72	2.01	39.21	25.98	77.03	7.84
Burundi	2016–17	6039	54.51	28.75	5.03	28.24	0.85	27.07	3.16	20.80	2.09
Cambodia	2021–22	3755	23.65	16.62	10.12	28.49	7.13	4.63	23.15	83.89	59.9
Cameroon	2018	4445	28.14	9.88	3.94	41.4	4.48	14.98	37.44	57.25	15.48
Chad	2014–15	9648	42.85	32.51	14.20	15.12	2.60	12.48	6.09	NA	NA
Comoros	2012	2354	27.74	14.61	11.68	55.22	5.55	32.57	47.72	NA	NA
Congo DR	2013–14	8014	44.09	23.20	7.91	27.29	5.15	19.00	10.92	NA	NA
Cote d'Ivoire	2021	4738	24.02	14.12	8.55	50.19	2.06	14.17	46.12	75.14	17.98
Egypt	2014	13,677	19.67	6.24	10.41	97.23	4.94	15.27	96.59	NA	NA
Gambia	2019–20	3805	18.08	12.17	5.12	69.1	2.79	40.73	49.90	75.68	50.53
Ghana	2022	4395	18.13	12.95	5.80	70.13	1.78	41.12	57.57	79.86	29.03
Guinea	2018	3371	30.73	15.66	8.60	41.53	1.98	32.12	19.65	65.97	8.02
India	2019–21	198,802	35.89	29.33	18.54	50.44	10.62	3.25	47.80	59.24	NA
Jordan	2012	6267	8.70	3.03	2.23	97.74	31.99	33.69	96.11	NA	NA
Kenya	2022	17,283	17.93	12.49	7.16	75.57	6.64	61.59	50.53	80.85	33.81
Kyrgyz Republic	2012	4000	17.77	3.55	2.90	91.42	36.03	25.76	89.93	NA	NA
Lesotho	2014	1312	34.60	11.20	3.51	59.87	11.34	55.11	19.52	NA	NA
Liberia	2019–20	2440	32.01	11.76	4.22	29.38	0.61	24.75	13.16	36.73	11.4
Malawi	2015–16	5110	35.23	11.55	3.05	34.72	6.06	29.38	7.59	29.41	2.32
Mali	2018	8223	26.55	18.61	9.44	57.22	1.98	45.22	38.39	53.00	8.22
Mozambique	2011	9313	39.26	13.09	5.19	48.52	5.67	41.95	17.28	NA	NA
Myanmar	2015–16	4186	30.46	18.54	6.62	59.05	9.71	19.20	51.56	NA	NA
Nepal	2022	2586	27.57	19.22	7.23	43.95	5.49	20.89	29.22	83.84	53.4
Niger	2012	4730	41.92	35.41	18.20	35.34	1.03	32.02	9.52	NA	NA
Nigeria	2018	11,314	36.06	21.21	6.63	43.92	3.90	31.61	31.29	54.54	9.37
Pakistan	2017–18	4095	38.22	21.88	7.91	51.2	4.51	3.39	48.76	36.29	10.78
Rwanda	2019–20	3806	33.34	7.51	1.13	59.04	6.00	56.69	14.89	43.93	6.71
Senegal	2019	5522	19.43	15.65	9.04	68.82	6.61	44.20	54.34	65.57	27.02
Sierra Leone	2019	4097	30.02	13.69	5.57	26.15	1.34	21.57	9.76	34.09	6.69
Tajikistan	2017	5844	18.28	7.92	6.49	85.04	12.56	11.22	83.76	50.62	8.99
Tanzania	2022	4791	28.82	11.27	3.63	40.15	6.04	30.17	22.86	56.86	7.32
Timor-Leste	2016	5557	46.73	40.02	24.04	39.2	4.54	12.05	34.99	66.71	12.46
Togo	2013–14	3161	28.41	16.83	7.31	53.9	2.72	44.36	29.00	NA	NA
Uganda	2016	4390	28.41	10.39	3.74	62.59	6.82	58.05	16.17	42.11	4.04
Yemen	2013	13,557	45.96	37.66	16.04	73.96	13.42	24.15	65.39	NA	NA
Zambia	2018	8681	34.74	11.88	4.07	45.53	6.46	31.55	27.51	45.41	5.92
Zimbabwe	2015	4914	25.25	7.47	3.48	50.88	11.61	36.15	24.72	68.34	15.6

*Note*. Summary statistics are based on weighted prevalence. The prevalence of mass media exposure was based on the pooled dataset (N = 439,639), mobile phone ownership was based on subsample A (N = 191,051), and internet access was based on subsample B (N = 152,812). Congo DR=Congo Democratic Republic. NA = Data is not available due to the absence of questionnaires on maternal mobile phone ownership and internet access.

### 3.1 Maternal media exposure and child anthropometric failure

**Fig. 2:**
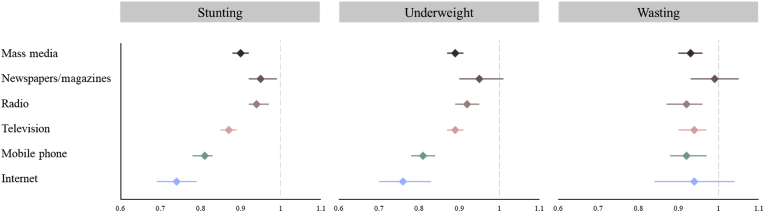
Adjusted odds ratios and 95% confidence intervals of child anthropometric failures by maternal media exposure across low- and middle-income countries *Note.* The association between maternal exposure to any and each type of mass media and child anthropometric failures was calculated using the pooled dataset (N = 439,639). The association between mobile phone ownership and internet access with child anthropometric was calculated based on subsample A (N = 191,051) and subsample B (N = 152,812), respectively. Each model adjusted for maternal age, marital status, age of child, sex of child, maternal education, household wealth, and type of residence.

In the fully adjusted model, children of mothers who were exposed to any mass media had 10% lower odds of stunting (OR = 0.90, 95% CI = 0.88–0.92), 11% lower odds of being underweight (OR = 0.89, 95% CI = 0.87–0.91), and 7% lower odds of wasting (OR = 0.93, 95% CI = 0.90–0.96) (Fig. 2). Mothers who read newspapers or magazines had 5% lower odds of their children being stunted (OR = 0.95, 95% CI = 0.92–0.99) than mothers who did not. However, there was no significant association between reading newspapers or magazines and being underweight and wasting. Those who listened to the radio had 6% lower odds of their children being stunted (OR = 0.94, 95% CI = 0.92–0.97), and 8% lower odds of being underweight (OR = 0.92, 95% CI = 0.89–0.95) and having wasting (OR = 0.92, 95% CI = 0.87–0.96), respectively. For mothers who watched television, the odds of their children being stunted, underweight, or wasting decreased by 13% (OR = 0.87, 95% CI = 0.85–0.89), 11% (OR = 0.89, 95% CI = 0.87–0.91), and 6% (OR = 0.94, 95% CI = 0.90–0.97), respectively. Those who owned mobile phones had 19% lower odds of children being stunted (OR = 0.81, 95% CI = 0.78–0.83) and underweight (OR = 0.81, 95% CI = 0.78–0.84) and 8% lower odds of their children having wasting (OR = 0.92, 95% CI = 0.88–0.97). Similarly, those who accessed the internet had a decreased probability of their children being stunted (OR = 0.74, 95% CI = 0.69–0.79), underweight (OR = 0.76, 95% CI = 0.70–0.83), and having wasting (OR = 0.94, 95% CI = 0.84–1.04).

### 3.2 Heterogeneity by maternal SES

**Fig. 3:**
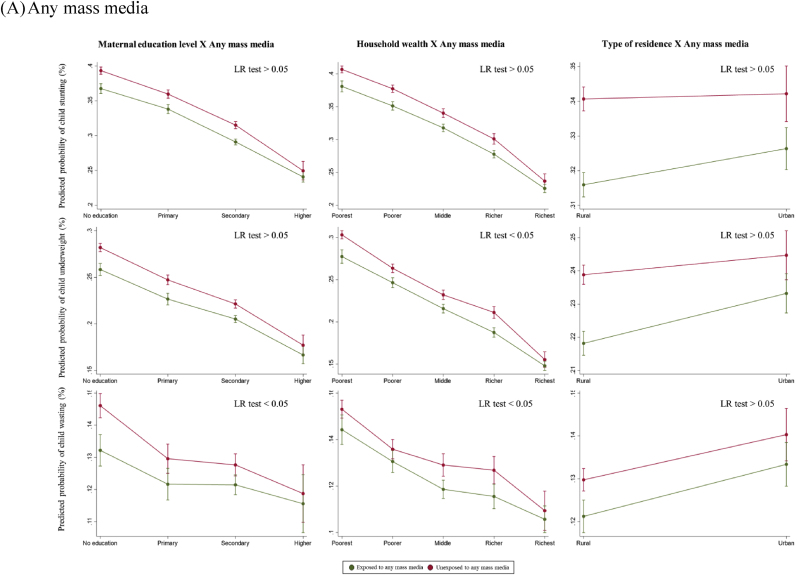

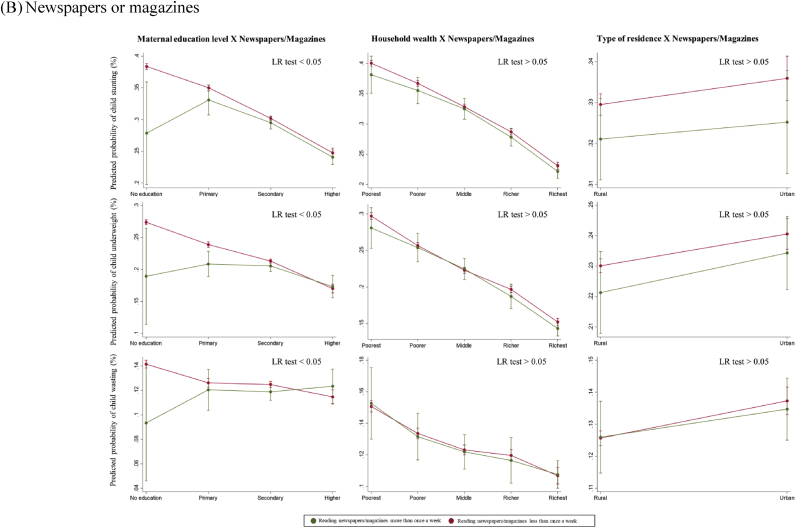

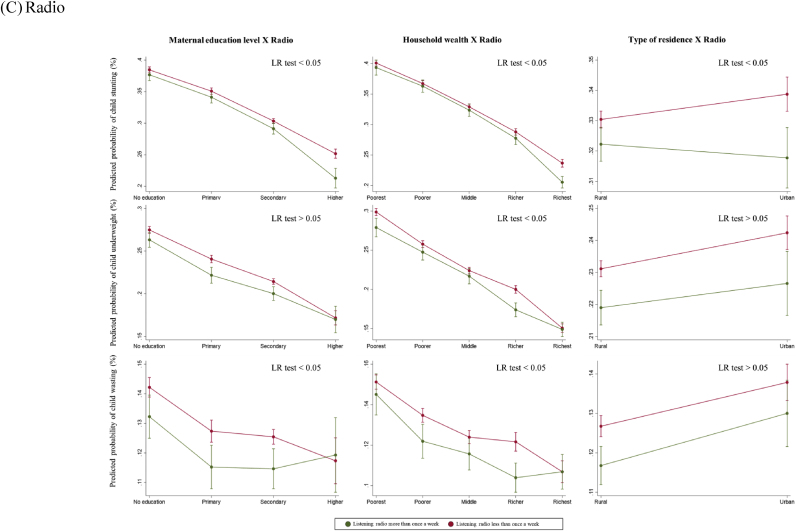

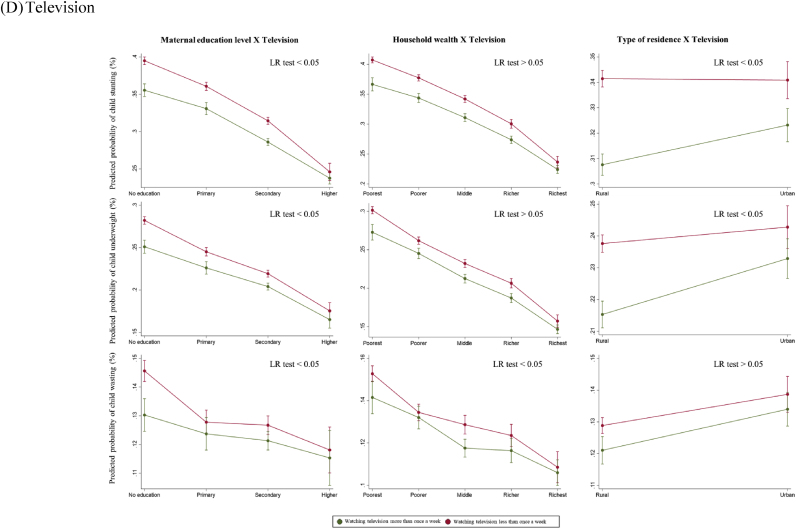
Predicted probabilities of child anthropometric failures as a function of maternal exposure to mass media and socioeconomic status *Note*. The marginal probabilities estimated were based on the pooled dataset (N = 439,639).

Overall, there was no statistically significant interaction between maternal exposure to any mass media and SES in respect to child anthropometric failures (LR test p > 0.05) (Fig. 3A). When assessed by each type of mass media, there was a statistically significant interaction in exposure to newspapers or magazines by maternal education level in terms of reducing the likelihood of all anthropometric failure outcomes (LR p < 0.05) (Fig. 3B). Among uneducated women, exposure to newspapers or magazines was associated with substantially lower probability of child stunting (28% vs. 38%), whereas this difference became minimal for secondary and higher education groups. A statistically significant interaction was observed between exposure to radio and household wealth quintiles in reducing the risk of child anthropometric failures (LR p < 0.05) (Fig. 3C). Among women from the richest quintiles, exposure to radio was associated with reduced likelihood of child stunting (21% vs. 24%). However, the difference was not statistically significant in the poorest, poorer, and middle quintiles. Lastly, there was a statistically significant interaction between exposure to television and maternal education level in reducing the probability of child anthropometric failure outcomes (LR p < 0.05) (Fig. 3D). Exposure to television was associated with lower probability of child stunting (36% vs. 40%) among uneducated women, while there was no difference among the higher education group.

**Fig. 4:**
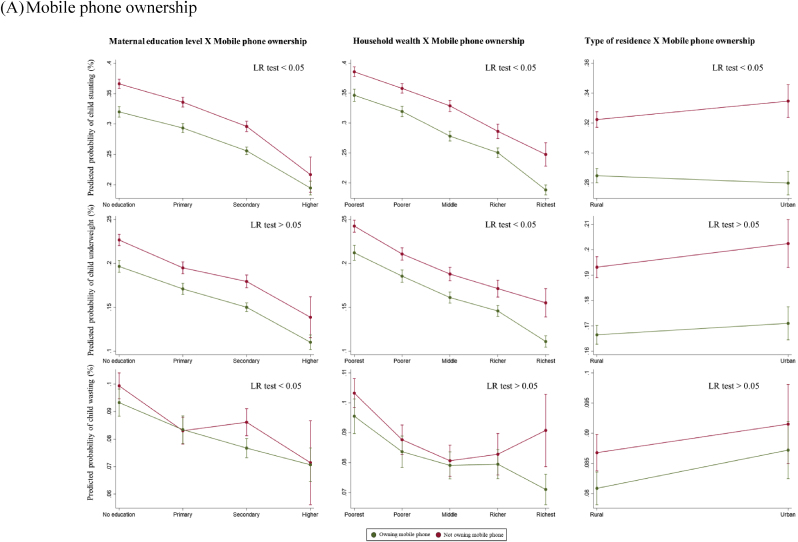

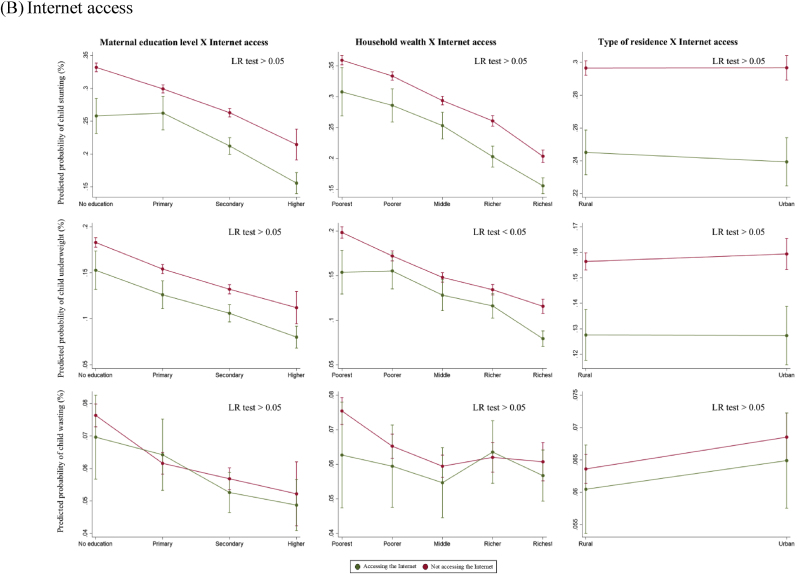
Predicted probabilities of child anthropometric failures as a function of maternal exposure to other types of media and socioeconomic status *Note*. The marginal probabilities estimated were based on subsample A (N = 191,051) and subsample B (N = 152,812) for mobile phone ownership and internet access respectively.

For the secondary outcomes, we found a statistically significant interaction in mobile phone ownership by maternal education level and household wealth quintiles (LR p < 0.05) (Fig. 4A). Among uneducated women, mobile phone ownership was significantly associated with lower child stunting (32% vs. 36%), but this difference was no longer significant for the higher education group. Similarly, among women from the richest quintile, mobile phone ownership was associated with a lower probability of child underweight (11% vs. 15%). However, the difference became minimal for women in middle and poorer quintiles. Fig. 4B showed no statistically significant difference in the association between mothers’ internet access and child anthropometric failures by maternal SES (LR p > 0.05).

### 3.3 Sensitivity analysis

Supplementary Fig. 1 shows the predicted probability of child anthropometric failures based on the frequency of maternal exposure to any mass media (not at all/less than once a week/at least once a week/daily). More frequent exposure to any mass media was associated with a lower predicted probability of child anthropometric failures: child stunting was 42% (95% CI: 41.7%–42.5%) when mothers did not utilize any mass media at all, 35% (95% CI: 34.2%–35.2%) when exposed to any mass media less than once a week, and 27% (95% CI: 27.2%–27.8%) when exposed to any mass media at least once a week. In the country-specific analyses, we found a consistent association between maternal mass media exposure and child anthropometric failures (Supplementary Fig. 2). For instance, the statistically significant relationship between child stunting and maternal exposure to mass media ranged from OR = 0.78 (95% CI = 0.66–0.91) in Chad to OR = 0.89 (95% CI = 0.86–0.92) in India. Lastly, the sensitivity analysis excluding country surveys affected by COVID-19 resulted in consistent findings, with the exception for child wasting for which a larger effect size was observed (Supplementary Table 3).

## 4 Discussion

This study used the latest nationally representative data across 40 LMICs and found several important findings. First, there was a significant relationship between maternal mass media exposure, mobile phone ownership, and internet access in lowering the odds of child anthropometric failures, even after adjusting for a comprehensive set of demographic and socioeconomic characteristics. This supports the potential role of media in inducing health behaviors and improving health outcomes by disseminating and educating health-related information to the general population. Similarly, previous studies have consistently found maternal media exposure, such as radio, television, and mobile messages, to be associated with better child-feeding practices (Jerin et al., 2020; Kim et al., 2019; Menon et al., 2016; Saaka et al., 2021), improved WASH-related knowledge and behaviors (Hanson et al., 2020), and child vaccination (Sohn et al., 2018) in LMICs. On the other hand, findings on the effect of maternal media exposure on child anthropometric failure outcomes remained rather mixed (George et al., 2021; Huo et al., 2022; Kim et al., 2019; Menon et al., 2016; Saaka et al., 2021; Young et al., 2021). In this context, our study supports that various types of media exposure, mobile phone ownership, and internet access can lower the risk of chronic and acute forms of child anthropometric failures.

Second, based on our interaction analyses, we found that the association between media exposure and child anthropometric failures is not necessarily homogeneous across all socioeconomic groups. In particular, maternal exposure to newspapers or magazines, television, and ownership of mobile phones significantly reduced the risk of child anthropometric failures among uneducated women but not for higher educated groups. Such interaction was also found in a previous study from sub-Saharan Africa, where the effects of mobile phone ownership in utilizing contraceptives and safe delivery were greater for less educated women, indicating that mobile phones can reduce knowledge gaps and allow access to health-related information more effectively (Iacoella et al., 2022). While women with higher education tend to have various channels to access health information, less educated women with limited opportunities may rely more exclusively on media to acquire knowledge that benefits their children’s health (Nordlund, 1978). By accessing information through media, less educated women may improve their health literacy (Jiang et al., 2024), thereby enhancing their children's nutrition.

At the same time, mothers’ radio exposure and mobile phone ownership were associated with reduced risk of anthropometric failures only among children from richer and richest quintiles. This heterogeneity can be partly explained by the fact that wealthier households have access to resources that enable them to translate the acquired health knowledge to health-inducing actions (Wardle & Steptoe, 2003). In doing so, media-based public health interventions can unintentionally exacerbate the existing health disparities by SES (Davidson et al., 2006).

Our findings have several important policy implications. The significant associations between various media types and child anthropometric failure call for more investments in media-based interventions to disseminate child-feeding and vaccination-related information to mothers. For example, mass media-based campaigns through television broadcasts (Beckstead et al., 2022; Kim et al., 2019; Menon et al., 2016; Rawat et al., 2017) and radio drama (Beckstead et al., 2022; Saaka et al., 2021) can convey appropriate complementary child-feeding practices (e.g., minimum dietary diversity for children) or WASH-related information to the audience, while mobile-based interventions can target more personalized messages (George et al., 2021) and calling (Jerin et al., 2020; Young et al., 2021). To date, media-based interventions for child undernutrition have been implemented at small scale (Jerin et al., 2020; Saaka et al., 2021; Young et al., 2021). Future programs should consider scaling up the media-based interventions, especially in countries with a high prevalence of child undernutrition.

At the same time, media-based health interventions should consider the complex interplay with SES. For instance, our study found that the association between maternal media exposure and the reduced risk of child undernutrition was more pronounced among uneducated women compared to higher education groups. In contrast, the association was greater among wealthier households compared to poorer households. These seemingly contradictory results indicate that while media exposure can be an important source of disseminating health-related information for uneducated women, it also poses a risk of exacerbating health inequities by disproportionately benefiting households with higher overall living standards. To prevent such unexpected consequences, media-based interventions should be complemented by broader financial and environmental support and practical advice for disadvantaged households, empowering them to perform appropriate health behaviors. A previous randomized controlled trial in Bangladesh emphasized that the intervention group with intensive media exposure required additional expenditures to achieve adequate diets for themselves and for their children (Warren et al., 2020). Another experimental study indicated that media-based interventions can significantly reduce the probability of child diarrhea if soaps and hygienic supplies were provided simultaneously (Reher et al., 2022). Consequently, media interventions that aim to help economically vulnerable mothers learn appropriate child nutrition knowledge should be supported with broader access to clean water, fresh food, and practical solutions.

This study should be interpreted in light of a few important data-related limitations. First, data from the cross-sectional study design hindered us from establishing causality in the observed associations. There might be unmeasured or residual confounding, such as environmental food insecurity, availability, and quality of healthcare services, which might contribute to child undernutrition. Future studies should address this by collecting more details about possible confounders. Second, there might be reporting bias since mothers self-reported their responses on media exposure and most covariates. However, the short reference period for questions on mass media and internet usage (i.e., limited to the last week and last month) minimizes the concerns around recall bias. If women over-reported their mass media exposure due to social desirability bias, then our findings may be overestimated. Third, there might be selection bias in our findings attributable to the inclusion criteria of the analytic sample. For instance, we excluded children missing data on primary exposure and outcome, and those who did not have information on any of the demographic and socioeconomic characteristics. However, the comparison between the final study sample and the original sample exhibited minor differences in demographic and socioeconomic characteristics, indicating a low likelihood of selection bias.

Fourth, measurements on maternal media exposure lacked details in terms of frequency and specific contents women had access to, thereby limiting our ability to explore the exact mechanisms through which maternal media exposure influences child anthropometric failure outcomes. Despite this limitation, previous studies have demonstrated that media exposure could lead to improvements in healthy behaviors (e.g., appropriate child-feeding practices (Beckstead et al., 2022; Suresh et al., 2019) and WASH-related behaviors (Alexander et al., 2019; Hanson et al., 2020)), aligning with our findings that media exposure can enhance health outcomes regardless of the specific content.

Fifth, this study was unable to control for contextual factors that may influence maternal media exposure and child anthropometric failure, such as the availability of infrastructure for media broadcasting and internet networks, better healthcare facilities and food security, and social support from communities. For instance, better social support from communities might strengthen women’s ability to perform healthy behaviors (e.g., appropriate child vaccination) based on the information received through media and lead to better child nutrition outcomes (Surkan et al., 2012). On the other hand, poor media infrastructure might hinder women’s media utilization and indicate poor environments for child nutrition in general (Wilkin, 2013). Although we attempted to adjust for some geographical disparities by controlling for the type of residence, future studies should investigate these contextual factors and their potential cross-level interactions with media exposure.

Lastly, this study included data collected during the COVID-19 pandemic for several countries: Burkina Faso, Cambodia, Cote d’Ivoire, Gambia, Ghana, India, Liberia, Nepal, and Rwanda. During the COVID-19 pandemic, health services disruptions, economic crisis, and breakdown of food markets could have increased child anthropometric failures in LMICs (Fahriani et al., 2021; Moynihan et al., 2021; Osendarp et al., 2021; Zhu et al., 2022). However, a recent study from India reported that child nutrition outcomes remained constant or only minimally worsened after the outbreak (Ko et al., 2023). Moreover, our sensitivity analysis excluding the nine countries suggests minimal impact of the COVID-19 pandemic on our main results.

## 5 Conclusion

This study underscores the crucial role of maternal exposure to mass media, mobile phones, and the internet in reducing child anthropometric failure across LMICs. The robust association between media exposure and child anthropometric failures, even after adjusting for demographic and socioeconomic factors, indicates media as a vital platform for health information dissemination. Moreover, the impact of specific media types varied by maternal SES, suggesting that targeted interventions are necessary. Conveying health-related information through media channels can be a valuable short-term strategy to reduce the risk of growth failures in early childhood. At the same time, more structural improvements are required to sustain these efforts equitably in the long run.

## CRediT authorship contribution statement

**Sohee Jung:** Writing – review & editing, Writing – original draft, Investigation, Formal analysis, Data curation. **Hwa-Young Lee:** Writing – review & editing, Investigation. **Seung-ah Choe:** Writing – review & editing, Investigation. **Hannah Oh:** Writing – review & editing, Investigation. **S.V. Subramanian:** Writing – review & editing, Investigation. **Rockli Kim:** Writing – review & editing, Supervision, Investigation, Conceptualization.

## Ethical statement

The DHS data are not collected specifically for this study and no one on the study team has access to identifiers linked to the data. These activities do not meet the regulatory definition of human subject research. As such, an Institutional Review Board (IRB) review is not required.

## Data availability

DHS data are available at https://dhsprogram.com (requiring a simple application).

## Funding

None.

## Declarations of interest

None.

## Data Availability

The authors do not have permission to share data.
