# Supplementary material for: Maternal media exposure and child anthropometric failures across 40 low- and middle-income countries
Source: SSM Popul Health. 2024 Dec 31;29:101746. doi: 10.1016/j.ssmph.2024.101746 (PMC11791352; doi:10.1016/j.ssmph.2024.101746)
Supplement: Multimedia component 1 [file mmc1.docx]

**SUPPLEMENTARY FILE**

Supplementary Table 1 Comparison of the original sample and final study sample by demographic and socioeconomic characteristics

|  | Original sample  (N=659,458) | Final study sample  (N=439,639) |
| --- | --- | --- |
|  | N (%) | |
| **Maternal age** |  |  |
| 15-19 years | 28,598 (4.34%) | 17,014 (3.87%) |
| 20-34 years | 509,738 (77.30%) | 348,272 (79.22%) |
| 35-49 years | 121,122 (18.37%) | 74,353 (16.91%) |
| **Marital status** |  |  |
| Not married | 98,534 (14.94%) | 49,832 (11.33%) |
| Married | 560,924 (85.06%) | 389,807 (88.67%) |
| **Child's age** |  |  |
| 0-24 months | 272,077 (41.26%) | 182,380 (41.48%) |
| 25-60 months | 387,381 (58.74%) | 257,259 (58.52%) |
| **Sex of child** |  |  |
| Male | 337,597 (51.19%) | 223,950 (50.94%) |
| Female | 321,861 (48.81%) | 215,689 (49.06%) |
| **Maternal education** | |  |
| No education | 210,658 (31.95%) | 128,622 (29.26%) |
| Primary | 155,578 (23.59%) | 93,718 (21.32%) |
| Secondary | 235,486 (35.71%) | 173,209 (39.40%) |
| Higher | 57,702 (8.75%) | 44,090 (10.03%) |
| **Household wealth** |  |  |
| Poorest | 167,089 (25.34%) | 111,497 (25.36%) |
| Poorer | 146,488 (22.21%) | 97,768 (22.24%) |
| Middle | 131,330 (19.91%) | 87,399 (19.88%) |
| Richer | 117,379 (17.80%) | 78,807 (17.93%) |
| Richest | 97,172 (14.74%) | 64,168 (14.60%) |
| **Type of residence** |  |  |
| Rural | 474,470 (71.95%) | 321,280 (73.08%) |
| Urban | 184,988 (28.05%) | 118,359 (26.92%) |

*Note*. Summary statistics are based on weighted prevalences.

Supplementary Table 2 Distribution of child anthropometric failures by demographic and socioeconomic characteristics and maternal mass media exposure across low- and middle-income countries

|  | Stunting, N (%) | | | | |  | Underweight, N (%) | |  | Wasting, N (%) | |  |
| --- | --- | --- | --- | --- | --- | --- | --- | --- | --- | --- | --- | --- |
|  | No | | Yes | | | *p-value* | No | Yes | *p-value* | No | Yes | *p-value* |
|  | N=294,411 | | N=145,228 | | |  | N=339,444 | N=100,195 |  | N=383,722 | N=55,917 |  |
| **Maternal age** | | <0.01 | | | | | <0.01 | | | <0.01 | | |
| 15-19 years | 11,427  (3.96) | | 5,587  (3.98) | | |  | 13,339 (3.99) | 3,675 (3.90) |  | 14,916 (3.96) | 2,098 (4.06) |  |
| 20-34 years | 232,834 (79.91) | | 115,438 (80.24) | | |  | 267,346 (79.55) | 80,926 (81.55) |  | 302,209 (79.56) | 46,063 (83.10) |  |
| 35-49 years | 50,150 (16.13) | | 24,203 (15.77) | | |  | 58,759 (16.45) | 15,594 (14.55) |  | 66,597 (16.48) | 7,756 (12.84) |  |
|  |  | |  | | |  |  |  |  |  |  |  |
| **Marital status** | | | ≥0.05 | | | |  | <0.01 | |  | <0.01 | |
| Not married | 33,418 (11.36) | | 16,414 (11.26) | | |  | 41,670 (12.35) | 8,162 (7.95) |  | 46,540 (12.15) | 3,292 (5.76) |  |
| Married | 260,993 (88.64) | | 128,814 (88.74) | | |  | 297,774 (87.65) | 92,033 (92.05) |  | 337,182 (87.85) | 52,625 (94.24) |  |
|  |  | |  | | |  |  |  |  |  |  |  |
| **Age of child** | | | <0.01 | | | |  | <0.01 | |  | <0.01 | |
| 0-24 months | 130,395 (44.43) | | 51,985 (36.07) | | |  | 145,500 (43.07) | 36,880 (37.02) |  | 155,348 (40.62) | 27,032 (48.75) |  |
| 25-60 months | 164,016 (55.57) | | 93,243 (63.93) | | |  | 193,944 (56.93) | 63,315 (62.98) |  | 228,374 (59.38) | 28,885 (51.25) |  |
|  |  | |  | | |  |  |  |  |  |  |  |
| **Sex of child** | | | <0.01 | | | |  | <0.01 | |  | <0.01 | |
| Male | 146,399 (49.92) | | 77,551 (53.35) | | |  | 170,859 (50.51) | 53,091 (52.85) |  | 193,669 (50.58) | 30,281 (54.26) |  |
| Female | 148,012 (50.08) | | 67,677 (46.65) | | |  | 168,585 (49.49) | 47,104 (47.15) |  | 190,053 (49.42) | 25,636 (45.74) |  |
|  |  | |  | | |  |  |  |  |  |  |  |
| **Maternal education** | | | <0.01 | | | |  | <0.01 | |  | <0.01 | |
| No education | 76,591 (24.69) | | 52,031 (35.34) | | |  | 90,803 (25.68) | 37,819 (36.60) |  | 110,514 (27.82) | 18,108 (30.91) |  |
| Primary | 59,859 (20.25) | | 33,859 (23.37) | | |  | 73,534 (21.67) | 20,184 (20.00) |  | 84,399 (21.98) | 9,319 (16.56) |  |
| Secondary | 122,519 (41.87) | | 50,690 (34.84) | | |  | 136,962 (40.44) | 36,247 (36.59) |  | 149,876 (39.21) | 23,333 (41.84) |  |
| Higher | 35,442 (13.19) | | 8,648  (6.44) | | |  | 38,145 (12.21) | 5,945 (6.81) |  | 38,933 (11.00) | 5,157 (10.69) |  |
|  |  | |  | | |  |  |  |  |  |  |  |
| **Household wealth** | | | <0.01 | | | |  | <0.01 | |  | <0.01 | |
| Poorest | 65,400 (19.81) | | 46,097 (29.76) | | |  | 77,432 (20.49) | 34,065 (31.72) |  | 94,450 (22.39) | 17,047 (27.89) |  |
| Poorer | 61,480 (19.83) | | 36,288 (24.45) | | |  | 73,118 (20.45) | 24,650 (24.35) |  | 84,998 (21.21) | 12,770 (22.31) |  |
| Middle | 59,204 (20.19) | | 28,195 (19.78) | | |  | 68,629 (20.37) | 18,770 (19.01) |  | 76,826 (20.21) | 10,573 (18.97) |  |
| Richer | 57,284 (20.79) | | 21,523 (15.96) | | |  | 64,521 (20.32) | 14,286 (15.45) |  | 69,732 (19.44) | 9,075 (17.52) |  |
| Richest | 51,043 (19.39) | | 13,125 (10.06) | | |  | 55,744 (18.37) | 8,424 (9.47) |  | 57,716 (16.75) | 6,452 (13.31) |  |
|  |  | |  | | |  |  |  |  |  |  |  |
| **Type of residence** | | | <0.01 | | | |  | <0.01 | |  | <0.01 | |
| Rural | 206,257 (67.09) | | 115,023 (76.81) | | |  | 240,699 (68.12) | 80,581 (77.54) |  | 277,881 (69.77) | 43,399 (73.94) |  |
| Urban | 88,154 (32.91) | | 30,205 (23.19) | | |  | 98,745 (31.88) | 19,614 (22.46) |  | 105,841 (30.23) | 12,518 (26.06) |  |
|  |  | |  | | |  |  |  |  |  |  |  |
| **Any mass media exposure** | | | | <0.01 | | |  | <0.01 | |  | <0.01 | |
| No | 131,677 (42.65) | | 83,487 (55.95) | | |  | 155,777 (43.88) | 59,387 (57.52) |  | 184,591 (46.21) | 30,573 (52.73) |  |
| Yes | 162,734 (57.35) | | 61,741 (44.05) | | |  | 183,667 (56.12) | 40,808 (42.48) |  | 199,131 (53.79) | 25,344 (47.27) |  |
|  |  | |  | | |  |  |  |  |  |  |  |
| **Reading newspapers/magazines** | | | | | <0.01 | |  | <0.01 | |  | <0.01 | |
| No | 268,143 (90.37) | | 136,965 (93.99) | | |  | 310,414 (90.85) | 94,694 (93.91) |  | 353,122 (91.49) | 51,986 (92.03) |  |
| Yes | 26,268 (9.63) | | 8,263  (6.01) | | |  | 29,030 (9.15) | 5,501  (6.09) |  | 30,600  (8.51) | 3,931 (7.97) |  |
|  |  | |  | | |  |  |  |  |  |  |  |
| **Listening to the radio** | | | | | <0.01 | |  | <0.01 | |  | <0.01 | |
| No | 237,791 (80.23) | | 124,407 (85.21) | | |  | 273,400 (79.86) | 88,798 (88.55) |  | 311,860 (80.67) | 50,338 (90.10) |  |
| Yes | 56,620 (19.77) | | 20,821 (14.79) | | |  | 66,044 (20.14) | 11,397 (11.45) |  | 71,862 (19.33) | 5,579  (9.90) |  |
|  |  | |  | | |  |  |  |  |  |  |  |
| **Watching a television** | | | | | <0.01 | |  | <0.01 | |  | <0.01 | |
| No | 160,297 (52.37) | | 97,226 (65.65) | | |  | 190,719 (54.29) | 66,804 (64.93) |  | 223,630 (56.49) | 33,893 (58.60) |  |
| Yes | 134,114 (47.63) | | 48,002 (34.35) | | |  | 148,725 (45.71) | 33,391 (35.07) |  | 160,092 (43.51) | 22,024 (41.40) |  |

*Note.* Summary statistics are based on weighted prevalence. P-value is signified at levels lower than 0.01 and 0.05 from two-sample chi-squared tests.

Supplementary Table 3 Sensitivity analysis on the association between maternal mass media exposure and child anthropometric failures after excluding data collected during the COVID-19 pandemic (2020-2022) (N=208,893)

|  |  | Stunting |  | Underweight | | Wasting |
| --- | --- | --- | --- | --- | --- | --- |
|  |  | OR (95% CI) | | | | |
| **Mass media Exposure** | | |  |  |  |  |
| Yes |  | 1 (base) |  | 1 (base) |  | 1 (base) |
| No |  | 0.90 (0.88-0.93)** | | 0.85 (0.82-0.88)** | | 0.83 (0.79-0.88)** |

*Note*. For the sensitivity analysis, data from Burkina Faso, Cambodia, Cote d’Ivoire, Gambia, Ghana, India, Liberia, Nepal, and Rwanda were excluded. Each model adjusted for maternal age, marital status, age of child, sex of child, maternal education, household wealth, and type of residence. P-value is signified at level lower than 0.05 with *, 0.01 with **.

Supplementary Figure 1 Predicted probabilities of child anthropometric failures by frequency of maternal exposure to any mass media

*Note*. Frequency of any mass media exposure on a weekly basis was categorized as follows: “not at all” if women hadn’t used any type of media, “less than once a week” if they used at least one type less than once, “at least once a week” if they used at least one type once or more than once, and “almost every day” if they used at least one type daily. The marginal probabilities estimated were based on the pooled dataset (N=439,639).


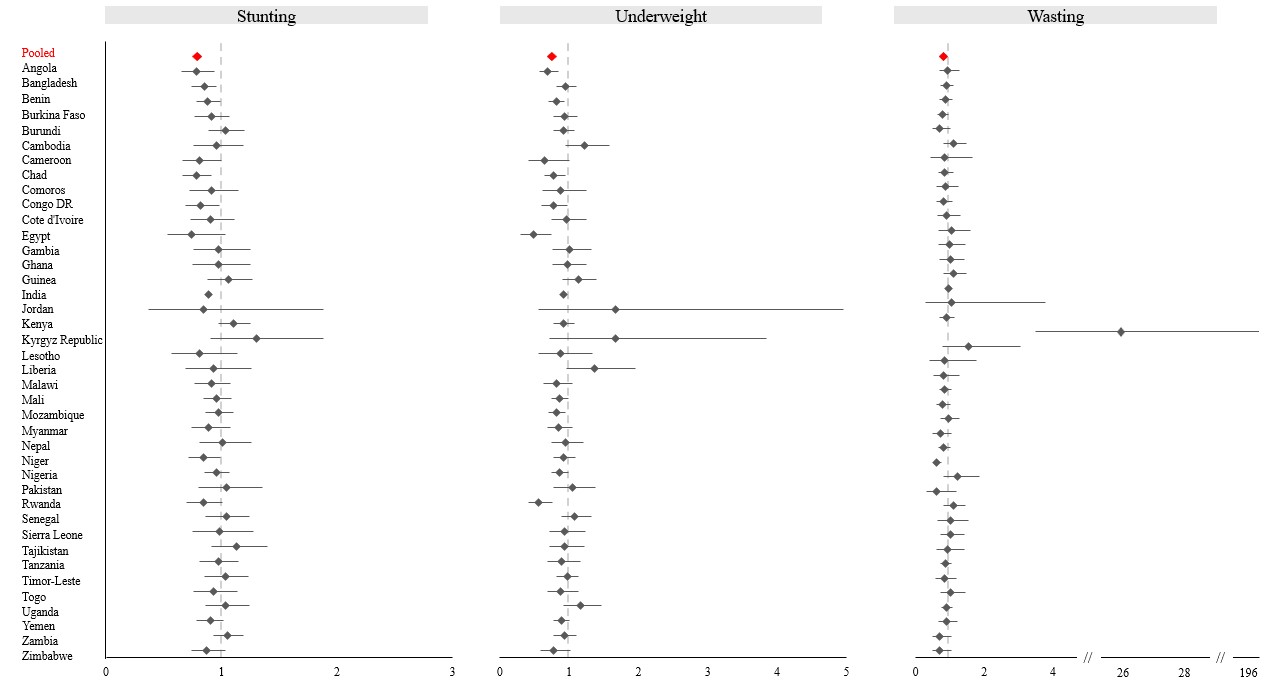
Supplementary Figure 2 Adjusted odds ratios and 95% confidence intervals of child anthropometric failures by maternal mass media exposure across 40 LMICs

*Note.* The association between maternal exposure to any mass media and child anthropometric failures was calculated using the pooled dataset (N=439,639) and each country dataset. Each model adjusted for maternal age, marital status, age of child, sex of child, maternal education, household wealth, and type of residence.
